# Health and Financial Risk-Taking Propensity During the COVID-19 Pandemic: Differences by Age and Time

**DOI:** 10.1093/geroni/igab046.3400

**Published:** 2021-12-17

**Authors:** Barış Sevi, Natalie Shook

**Affiliations:** University of Connecticut, Storrs, Connecticut, United States

## Abstract

The COVID-19 pandemic has presented a global health threat of unprecedented magnitude and had a devastating impact on the world’s economy. Accordingly, the riskiness of decisions related to health and finance may have increased. However, health and financial threats have differentially affected different age groups. For example, COVID-19 posed a greater health threat to older adults (65+ years) than younger or middle-aged adults, whereas financial threat due to the pandemic affected younger and middle-aged adults more than older adults. This study examined differences in the levels of health and financial risk-taking propensity by time of the pandemic and age group: young (18-39 years), middle-aged (40-64 years), and older adults (65+ years). A sample of 488 individuals residing in the US (245 Woman; Mage = 51.07, SD = 15.99) completed three waves of surveys in March, April, and May 2020. We found that risk-taking propensity for both health and financial decisions decreased over time. The risk-taking propensity was significantly lower in April and May than March, but risk-taking propensity in April and May did not significantly differ. The three age groups were all significantly different than each other in both health and financial risk-taking propensity at all three waves. Younger adults reported higher risk-taking propensity than older and middle-aged adults, and middle-aged adults reported higher risk-taking propensity than older adults. The findings indicate that the pandemic may have influenced all individuals to take less risks in the fields of health and finance regardless of their age.

